# Selective disrupted gray matter volume covariance of amygdala subregions in schizophrenia

**DOI:** 10.3389/fpsyt.2024.1349989

**Published:** 2024-04-29

**Authors:** Zhongyu Chang, Liping Liu, Liyuan Lin, Gang Wang, Chen Zhang, Hongjun Tian, Wei Liu, Lina Wang, Bin Zhang, Juanjuan Ren, Yu Zhang, Yingying Xie, Xiaotong Du, Xiaotong Wei, Luli Wei, Yun Luo, Haoyang Dong, Xin Li, Zhen Zhao, Meng Liang, Congpei Zhang, Xijin Wang, Chunshui Yu, Wen Qin, Huaigui Liu

**Affiliations:** ^1^ Department of Radiology and Tianjin Key Laboratory of Functional Imaging, Tianjin Medical University General Hospital, Tianjin, China; ^2^ Department of Psychiatry, The First Psychiatric Hospital of Harbin, Harbin, Heilongjiang, China; ^3^ Wuhan Mental Health Center, The Ninth Clinical School, Tongji Medical College, Huazhong University of Science and Technology, Wuhan, China; ^4^ Department of Biochemistry and Psychopharmacology, Shanghai Mental Health Center, Shanghai, China; ^5^ Department of Psychiatry, Tianjin Fourth Center Hospital, The Fourth Central Clinical College, Tianjin Medical University, Tianjin, China; ^6^ Department of Psychiatry, The First Affiliated Hospital of Harbin Medical University, Harbin, China; ^7^ School of Medical Imaging, Tianjin Medical University, Tianjin, China; ^8^ State Key Laboratory of Experimental Hematology, Tianjin Medical University General Hospital, Tianjin, China

**Keywords:** schizophrenia, amygdala subregions, gray matter volume, structural covariance, magnetic resonance imaging

## Abstract

**Objective:**

Although extensive structural and functional abnormalities have been reported in schizophrenia, the gray matter volume (GMV) covariance of the amygdala remain unknown. The amygdala contains several subregions with different connection patterns and functions, but it is unclear whether the GMV covariance of these subregions are selectively affected in schizophrenia.

**Methods:**

To address this issue, we compared the GMV covariance of each amygdala subregion between 807 schizophrenia patients and 845 healthy controls from 11 centers. The amygdala was segmented into nine subregions using FreeSurfer (v7.1.1), including the lateral (La), basal (Ba), accessory-basal (AB), anterior-amygdaloid-area (AAA), central (Ce), medial (Me), cortical (Co), corticoamygdaloid-transition (CAT), and paralaminar (PL) nucleus. We developed an operational combat harmonization model for 11 centers, subsequently employing a voxel-wise general linear model to investigate the differences in GMV covariance between schizophrenia patients and healthy controls across these subregions and the entire brain, while adjusting for age, sex and TIV.

**Results:**

Our findings revealed that five amygdala subregions of schizophrenia patients, including bilateral AAA, CAT, and right Ba, demonstrated significantly increased GMV covariance with the hippocampus, striatum, orbitofrontal cortex, and so on (permutation test, *P*< 0.05, corrected). These findings could be replicated in most centers. Rigorous correlation analysis failed to identify relationships between the altered GMV covariance with positive and negative symptom scale, duration of illness, and antipsychotic medication measure.

**Conclusion:**

Our research is the first to discover selectively impaired GMV covariance patterns of amygdala subregion in a large multicenter sample size of patients with schizophrenia.

## Introduction

Schizophrenia (SCZ), as a mental disorder with a high rate of disability, has a serious influence on the normal life of patients and their families, and imposes a significant burden on society (1). The main clinical symptoms of schizophrenia patients include hallucinations, disorganized thinking, impaired executive ability, and reduced emotional expression (2). Numerous neuroimaging studies of SCZ have demonstrated the abnormalities in multiple brain regions, especially the prefrontal cortex (PFC) (3) and hippocampus (4), and proposed a hypothesis that SCZ is a widespread dysconnection disorder (5). Recently, the amygdala has been a research focus in human mental health (6), and shows a close relationship with the pathophysiology of schizophrenia (7).

As a connecting hub, the amygdala has extensive connections to cortical and subcortical areas such as the frontal lobe, temporal lobe, and striatum (8), and plays important roles in emotional processing, memory encoding, and executive control functions (7). Research has highlighted that the medial prefrontal lobe, which exhibits significant connectivity with the amygdala, plays a crucial role in modulating stress, facilitating social cognition, making decisions in events, and regulating emotions (9–11). Furthermore, evidence suggests that negative psychological states, such as stress or anxiety, can impair the regulatory functions of the medial prefrontal-amygdala circuit (12, 13). In parallel, the amygdala’s direct and robust connection with the hippocampus is essential for processing contextual memories and behaviors (14–16). Investigations using structural MRI have revealed a reduction in amygdala volume in schizophrenia patients and their first-degree relatives compared to healthy subjects (17–19). Studies utilizing resting state functional MRI studies have identified disturbances in the amygdala’s functional connectivity with other cerebral regions (20–23). Such structural and functional anomalies contribute to cognitive deficits in schizophrenia (24, 25). Therefore, the disrupted amygdala connectivity may serve as a potential trigger for schizophrenia, and its intensive study contributes to our understanding of the pathophysiological mechanisms of schizophrenia (26).

The amygdala can be defined as a more detailed heterogeneous complex of nuclei due to its different cytoarchitecture, neurotransmitters, and connectivity patterns (27). It has been demonstrated that different heterogeneous nuclei are embedded in various connectivity pathways and perform roles in processing different cognitions. For instance, the cortical nucleus is the main projection nucleus of the olfactory cortex (27); the lateral nucleus has a significant role in the fear conditioning reflex (28); the central nucleus receives information input from the hippocampus (29). Reductions in the volume of refined amygdala nuclei were apparent in schizophrenia: all nuclei except the medial nucleus, suggesting a more widespread change in amygdala morphology than previously thought (30).

According to the dysconnection hypothesis, schizophrenia is characterized by a reduced ability to integrate information between distinct brain regions (5, 31, 32). Structural covariance is often used to analyze the topology of the brain as a method capable of testing for connectivity deficits in brain regions. Differences in internal covariance patterns between schizophrenia and healthy controls were revealed by calculating correlations in morphological indicators such as volume, cortical thickness, or surface area of brain regions (33). Recently, based on the more refined segmentation approach, researchers found that the amygdala subregions exhibit selective regional structural damages in schizophrenia patients (30, 34). Thus, we have developed a hypothesis that the gray matter volume (GMV) covariance of amygdala subregions would be selectively impaired in schizophrenia. To test this hypothesis, we investigated whether GMV covariance of amygdala subregions selectively differs between schizophrenia and healthy controls; moreover, we tried to validate if the impairment patterns could be replicated by multicenter datasets from multiple first-episode versus non-first-episode schizophrenia centers.

## Materials and methods

### Subjects

A total of 11 datasets was enrolled in the study as follows: 2 first-episode schizophrenia local datasets (FE_Guangzhou and FE_Harbin) and 9 non-first-episode schizophrenia local and public datasets with 5 local (NFE_Tianjin1, NFE_Tianjin2, NFE_Shanghai, NFE_Harbin and NFE_Wuhan) and 4 public datasets (NFE_BrainGluSchi [www.schizconnect.org], NFE_COBRE [http://fcon_1000projects.nitrc.org/indi/retro/cobre.html], NFE-NMorphCH [www.schizconnect.org] and NFE_UCLA [https://openneuro.org/datasets/ds000030/versions/00016]). The patients with schizophrenia were diagnosed by the structured clinical interview in DSM-IV. Exclusive criteria were contraindications to MRI; intracerebral lesions or structural abnormalities; no history of mental illness; no history of alcohol, drug, or substance abuse; female subjects were not pregnant or lactating. Other exclusive criteria for healthy controls were a history of any Axis I or II disorder and psychiatric disorders and first-degree relatives with psychiatric disorders. Our definition of a patient with first-episode schizophrenia was that the patient was not on antipsychotic medication or had not been on medication for more than two weeks at the time of inclusion in the trial. To eliminate possible effects of covariates on the results, we matched the age and sex of healthy controls (HC) to patients with schizophrenia in each center separately. Eventually, we included 831 schizophrenia patients and 851 healthy controls. The studies involving human participants were reviewed and approved by the Ethics Committee of Tianjin Medical University General Hospital, Tianjin, China. The patients/participants provided their written informed consent to participate in this study. Relevant Institutional Review Boards also approved the four public test-retest datasets, and detailed recruitment information was provided on the website.

### MRI data acquisition

During the acquisition, subjects were told to keep their bodies still, to immobilize their heads using comfortable and tight foam pads, and to use earplugs to reduce scanning noise. Structural MRI data were all acquired by 3.0T MRI scanners, including two GE MR750 scanners, four Siemens Trio Tim scanners, one Siemens Prisma, one Siemens Prisma_fit scanner, one Philips Ingenia, and one Philips Achiva scanner. A 3D fast spoided gradient echo sequence was used to acquire the high-resolution T1-weighted structural MRI images. The scanner information, sequence, and acquisition parameters are shown in **Table 1**. To ensure the image quality of the dataset, each MRI image was scrutinized by two experienced MRI experts (W.Q and H.L). We removed 30 subjects with obvious artifacts (head motion artifacts, wrap-around artifacts, metal artifacts, etc.), including 24 patients with schizophrenia and 6 healthy controls. Finally, the T1 structural MRIs of 1652 participants, including 807 schizophrenia patients and 845 healthy controls, were included in the following analyses.

**Table 1:** Scanning information for structural MRI in each Research Centers.

Center	Vendor	Model	Field	Sequence	TR/TE/TI (ms)	FA (°)	Matrix size
FE_Guangzhou	Philips	Achieva	3T	TFE	8.2/3.8/0	7	256 × 256
FE_Harbin	GE	MR750	3T	BRAVO	8.2/3.2/450	12	256 × 256
NFE_Tianjin1	GE	MR750	3T	BRAVO	8.2/3.2/450	12	256 × 256
NFE_Tianjin2	Siemens	Prisma	3T	MPRAGE	2000/2.3/900	8	256 × 256
NFE_Shanghai	Siemens	Prisma_fit	3T	MPRAGE	2000/2.3/900	8	256 × 256
NFE_Wuhan	Philips	Ingenia	3T	TFE	6.8/3.1/0	7	256 × 256
NFE_Harbin	GE	MR750	3T	BRAVO	8.2/3.2/450	12	256 × 256
NFE_BrainGluSchi	Siemens	Trio Tim	3T	MPRAGE	2530/1.6/1200	7	256 × 256
NFE_COBRE	Siemens	Trio Tim	3T	MPRAGE	2530/1.6/1200	7	256 × 256
NFE_NMorphCH	Siemens	Trio Tim	3T	MPRAGE	2400/3.2/1000	8	256 × 256
NFE_UCLA	Siemens	Trio Tim	3T	MPRAGE	2530/3.3/1100	7	256 × 256

TR, Repetition time; TE, Echo Time; TI, Inversion time; FA, Flip angle.

### Amygdala segmentation

Previous neuroimaging approaches divided the amygdala into 2-4 nuclei (35–38). However, most of these amygdala atlases are group-based and ignore the inter-subject variability of the subfields’ boundaries, which may introduce bias in calculating the covariance between these amygdala subfields and other brain regions. Thus, in this study, we turn to apply an individual-level segmentation atlas to define the amygdala subfields, which has been incorporated into the FreeSurfer software package v7.1.1 (http://surfer.nmr.mgh.harvard.edu/). This automatic atlas segments the amygdala subfields based on each person’s high-resolution MRI data and Bayesian inference algorithm with postmortem specimens at high resolution at 7T field strength as reference (39). The amygdala of each subject was automatically segmented into 9 subregions per hemisphere: lateral (La), basal (Ba), accessory-basal (AB), anterior-amygdaloid-area (AAA), central (Ce), medial (Me), cortical (Co), corticoamygdaloid-transition (CAT), paralaminar (PL) nucleus (39). Finally, we extracted the volume of each amygdala subregion and the total volume for each side of the amygdala.

### Gray matter volume calculation

Other cerebral areas’ absolute GMV and total intracranial volume (TIV) were obtained by the new segment pipeline using SPM12 (SPM12; http://www.fil.ion.ucl.ac.uk) with steps including bias correction, segmentation, spatial normalization using Diffeomorphic Anatomical Registration Through Exponentiated Lie Algebra (DARTEL) algorithms, Jacobian modulation, and smoothing with full width at half maximum (FWHM) kernel of 8×8×8mm^3^.

### Statistics for GMV covariance alterations pattern of amygdala subregions

In order to eliminate systematic deviations in the eleven center GMVs, combat harmonization model was carried out before statistics, in which the center IDs were defined as the batch variable, and group, age, sex and total intracranial volume (TIV) were considered as biological covariates (40, 41). Then, we employed a general linear model (GLM) to construct an interaction model between the group (schizophrenia vs. healthy controls) and the volume of the amygdala subregions. This model has been frequently used to estimate the structural covariance coefficient map of a seed region across subjects, and to compare intergroup differences in their covariance coefficient at the voxel level (42, 43). Specifically, the basic idea of the GLM for structural covariance comparison in the present study is to estimate the covariate coefficients between each amygdala subregion and each other brain voxels for schizophrenia patients and healthy control groups, respectively. Then, a two-sample t-test was employed to compare whether the covariate coefficients between the two groups differed, with age, sex, and total intracranial volume (TIV) as nuisance confounders [equation (1)]:


(1) $$GMV=\beta_{1}.SCZ+\beta_{2}.HC+\beta_{3}.VolS_{SCZ}+\beta_{4}.VolS_{HC}+\beta_{covs}.Covs$$

where *GMV* denotes the gray matter volume per voxel, *SCZ* and *HC* represent schizophrenia and healthy controls, respectively. *VolS* denotes the volume of the amygdala subregion, and *Covs* refers to covariates that need to be controlled for. *β_3_* and *β_4_* signify the covariate coefficients for schizophrenia and healthy controls. The effect is the difference between these covariate coefficients, expressed as *β_3_-β_4_*. The assessment of whether this covariate difference is significant was performed using a non-parametric permutation-based two-sample t-test (with 5,000 permutations) and further employing threshold-free cluster enhancement (*TFCE*) combined with family-wise error (*FWE*) correction to control for false positives due to multiple comparisons at the voxel level. To further correct the multiple comparisons false positives derived by multiple subregions, we evaluated the effective numbers of independent tests Meff (6.17 times) (44), and at last, using a strict threshold for the statistics of all amygdala subregions (*P*< 0.05 [TFCE-FWE threshold]/6.17 = 0.0081). To clarify whether there is an advantage by sub-regional analysis, we conducted the same GLM to compare the intergroup differences in GMV covariance of the whole left and right amygdala with the same significant threshold as the subregions. Finally, to validate the stability of the covariance pattern alterations in each of the 11 datasets, we extracted the average GMV of the brain with changed covariance in each amygdala subregion and carried out ROI-wise GLM described above for each site.

Furthermore, to better observe the alteration of subregional structure, we employed a GLM model to investigate whether there were GMV differences in whole amygdala and each of its subregions between schizophrenia patients and healthy controls within both male and female subgroups, taking age and TIV as confounding confounders (*P*< 0.0081). Additionally, we compared whether there were inter-sex differences between the observed differences in the male group and those observed in the female group.

### Target region definition and revealing subregion-specific covariance disruption patterns

To reveal the amygdala subregion-specific covariance disruption patterns of schizophrenia, we extract the average GMV of brain region-of-interest (ROI) with changed covariance in at least one amygdala subregion. This procedure was performed with the following steps: (1) for each subregion’s statistic map, we generated a binary mask where intergroup differences of the GMV covariance between this subregion and voxel within the mask survived; (2) we merged these binary masks of all amygdala subregion into a union mask; (3) we identified the intersection voxels between the union mask and each AAL3 region (45); (4) we calculated the overlapping ratio between the intersection voxels and each ALL region; (5) we selected the target ROIs with the criteria: the overlapping ratio is greater than 15% the volume of overlapping region is greater than 1mm^3^; (6) finally, the average GMV of each target ROIs was extracted for each subject.

We then carried out ROI-wise GLM to compare the intergroup differences in GMV covariance between each amygdala subregion and target ROI. We generated fingerprints plot to demonstrate the unique structural covariance disruption patterns between amygdala subregions in schizophrenia. This plot had been applied to represent the unique spatial connectivity patterns of a specific brain subregion that differentiated it from other subregions (46, 47). Besides, we conducted a sex-specific analysis, which could provide valuable insights into whether the observed GMV covariance disruptions are differed between the females or males. We further explored whether these abnormal GMV covariance patterns were replicable across different data centers.

### Association between the GMV covariance and clinical features

The clinical features included the Positive and Negative Syndrome Scale (PANSS), duration of illness, and dose of antipsychotic medication (chlorpromazine equivalent doses) (48). A Spearman correlation analysis was performed between the GMV covariance alterations and the clinical features (*P*< 0.05).

## Results

### Demographic characteristics

We finally included 807 schizophrenia patients (32.44 ± 10.34 years, 484 males) and 845 healthy controls (32.57 ± 10.47 years, 491 males). The detailed demographic information of the subjects is displayed in **Table 2**. There were no statistical group differences in age (F = 1.09, *P* = 0.79) or sex (**χ^2^** = 0.60, *P* = 0.44). There were no significant differences in either sex or age between schizophrenia patients and healthy controls in each center (*P* > 0.05).

**Table 2:** Demographic information of recruited datasets.

		SCZ	HC	Statistics	*P*
Total	Age(years)	32.44(10.34)	32.57(10.41)	F = 1.09	0.793
	Sex(M/F)	484/323	491/354	**χ^2^=**0.60	0.440
FE_Guangzhou	Age(years)	21.76(7.62)	20.13(6.00)	F = 4.44	0.145
	Sex(M/F)	44/40	41/30	**χ^2^=**0.45	0.504
FE_Harbin	Age(years)	31.62(8.66)	32.41(9.08)	F = 0.02	0.546
	Sex(M/F)	38/40	59/52	**χ^2^=**0.36	0.548
NFE_Tianjin1	Age(years)	34.01(9.86)	33.78(11.15)	F = 8.95	0.872
	Sex(M/F)	58/57	48/57	**χ^2^** =0.49	0.484
NFE_Tianjin2	Age(years)	34.22(8.92)	34.69(10.01)	F = 0.59	0.743
	Sex(M/F)	45/37	46/53	**χ^2^** =1.27	0.260
NFE_Shanghai	Age(years)	29.82(8.31)	28.73(7.40)	F = 0.28	0.538
	Sex(M/F)	15/18	32/17	**χ^2^** =3.18	0.075
NFE_Wuhan	Age(years)	32.44(6.87)	31.18(7.61)	F = 0.95	0.304
	Sex(M/F)	53/37	31/24	**χ^2^** =0.09	0.765
NFE_Harbin	Age(years)	32.57(10.88)	32.41(9.08)	F = 2.74	0.905
	Sex(M/F)	38/49	59/52	**χ^2^** =1.75	0.186
NFE_BrainGluSchi	Age(years)	34.74(12.47)	38.92(12.74)	F = 0.26	0.077
	Sex(M/F)	60/5	45/7	**χ^2^** =1.05	0.307
NFE_COBRE	Age(years)	36.75(12.48)	37.79(11.13)	F = 3.06	0.573
	Sex(M/F)	64/16	60/23	**χ^2^** =1.33	0.249
NFE_NMorphCH	Age(years)	32.70(7.32)	30.31(8.10)	F = 0.62	0.172
	Sex(M/F)	31/12	19/17	**χ^2^** =3.15	0.076
NFE_UCLA	Age(years)	36.46(8.88)	34.84	F = 0.04	0.315
	Sex(M/F)	38/12	51/22	**χ^2^** =0.56	0.455

Numerical variables are presented as means (standard deviations).

SCZ, Schizophrenia; HC, Healthy control; M/F, Male/Female; FE, First episode schizophrenia center; NFE, Non first episode schizophrenia center.

First episode schizophrenia center: FE_Guangzhou; FE_Harbin.

Non first episode schizophrenia center: NFE_Tianjin1; NFE_Tianjin2; NFE_Shanghai; NFE_Wuhan; NFE_Harbin; NFE_BrainGluSchi; NFE_COBRE; NFE_NMorphCH; NFE_UCLA.

### Altered GMV covariance patterns of amygdala subregions in schizophrenia

**Figure 1:**
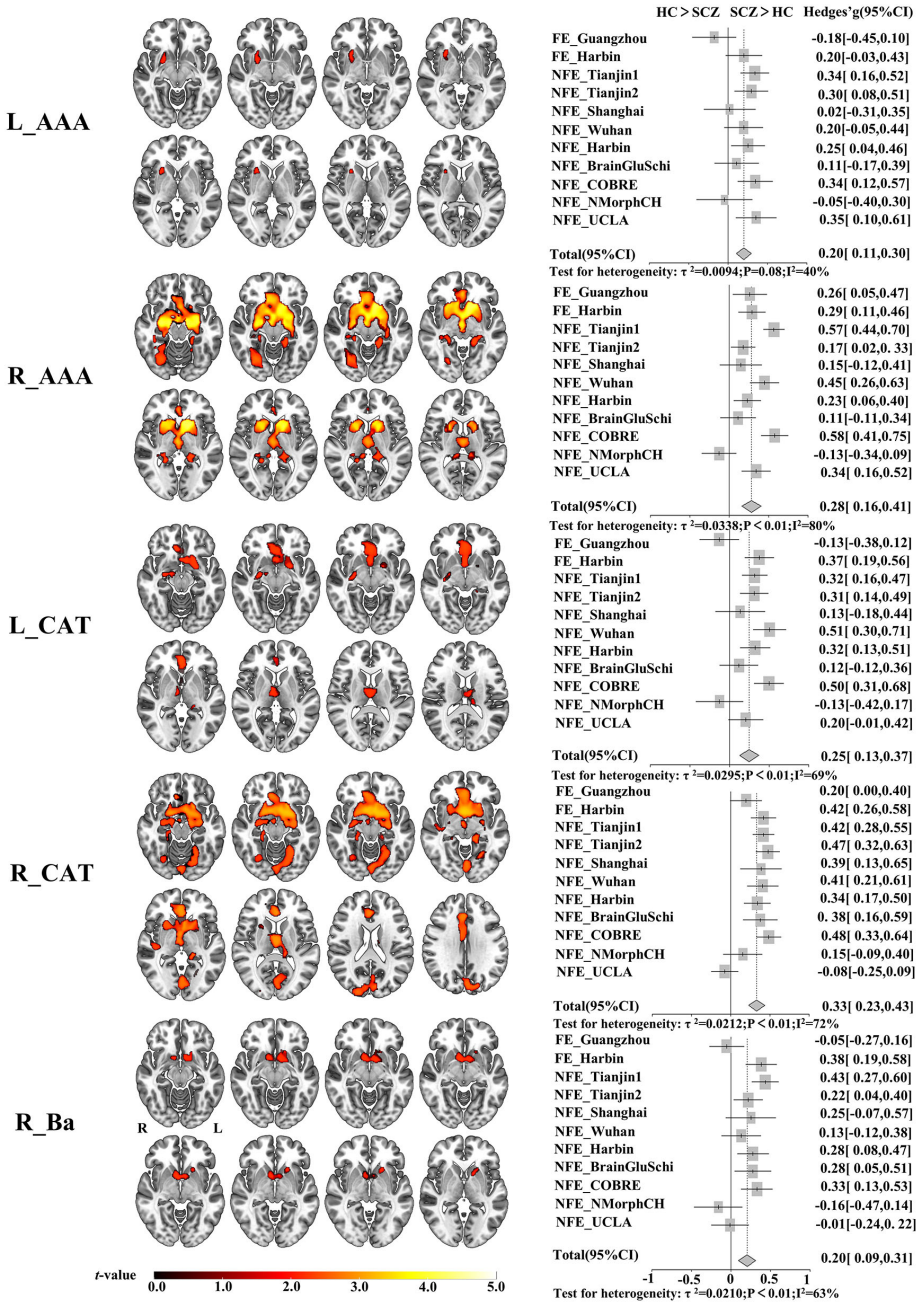
Altered GMV covariance patterns of the five amygdala subregions in patients with schizophrenia and the validation across centers. The color bar indicates the intensity of altered GMV covariance in schizophrenia (*t*-value). The forest plots represent the effect size (also known as Hedges’ adjusted g*) for inter-group GMV covariance differences of the five amygdala subregions in each centers. L_AAA, Anterior-amygdaloid-area of left hemisphere; R_AAA, Anterior-amygdaloid-area of right hemisphere; L_CAT, Corticoamygdaloid-transitio of left hemisphere; R_CAT, Corticoamygdaloid-transitio of right hemisphere; R_Ba, Basal nucleus of right hemisphere. L, left, R, right. SCZ, Schizophrenia, HC, Healthy control.

We found significantly increased GMV covariance patterns in five amygdala subregions, L_AAA, R_AAA, L_CAT, R_CAT and R_Ba (**Figure 1**). Among them, changed GMV covariance was found in both hemispheres of anterior-amygdaloid-area and corticoamygdaloid-transition with a relatively stable altered GMV covariance patterns. Both bisymmetric AAA subregions exhibited increased GMV covariance with the bilateral putamen. Additionally, the R_AAA subregion also showed increased GMV covariance with the bilateral caudate nucleus, hippocampus, and parahippocampal gyrus. The bilateral CAT subregions demonstrated the most extensive significant GMV covariance alterations, including the bilateral putamen, caudate nucleus, hippocampus, parahippocampal gyrus, olfactory cortex, anterior and middle cingulate cortex, fusiform gyrus, lingual gyrus, and superior frontal gyrus. Increased GMV covariance of R_Ba with bilateral accumbens nucleus and the left putamen.

Moreover, the trend of the altered GMV covariance patterns can be replicated in most centers, such as FE_Harbin, NFE_Tianjin1, NFE_Tianjin2, NFE_Shanghai, NFE_Wuhan, NFE_Harbin, NFE_BrainGluSchi, NFE_COBRE. Specifically, we validated the increased GMV covariance pattern of R_AAA except for the NFE_NMorphCH center and the pattern of R_CAT except for the NFE_UCLA center. However, the FE_Guangzhou and NFE_NMorphCH obtained opposite results in the subregions of L_AAA, L_CAT, and R_Ba.

**Figure 2:**
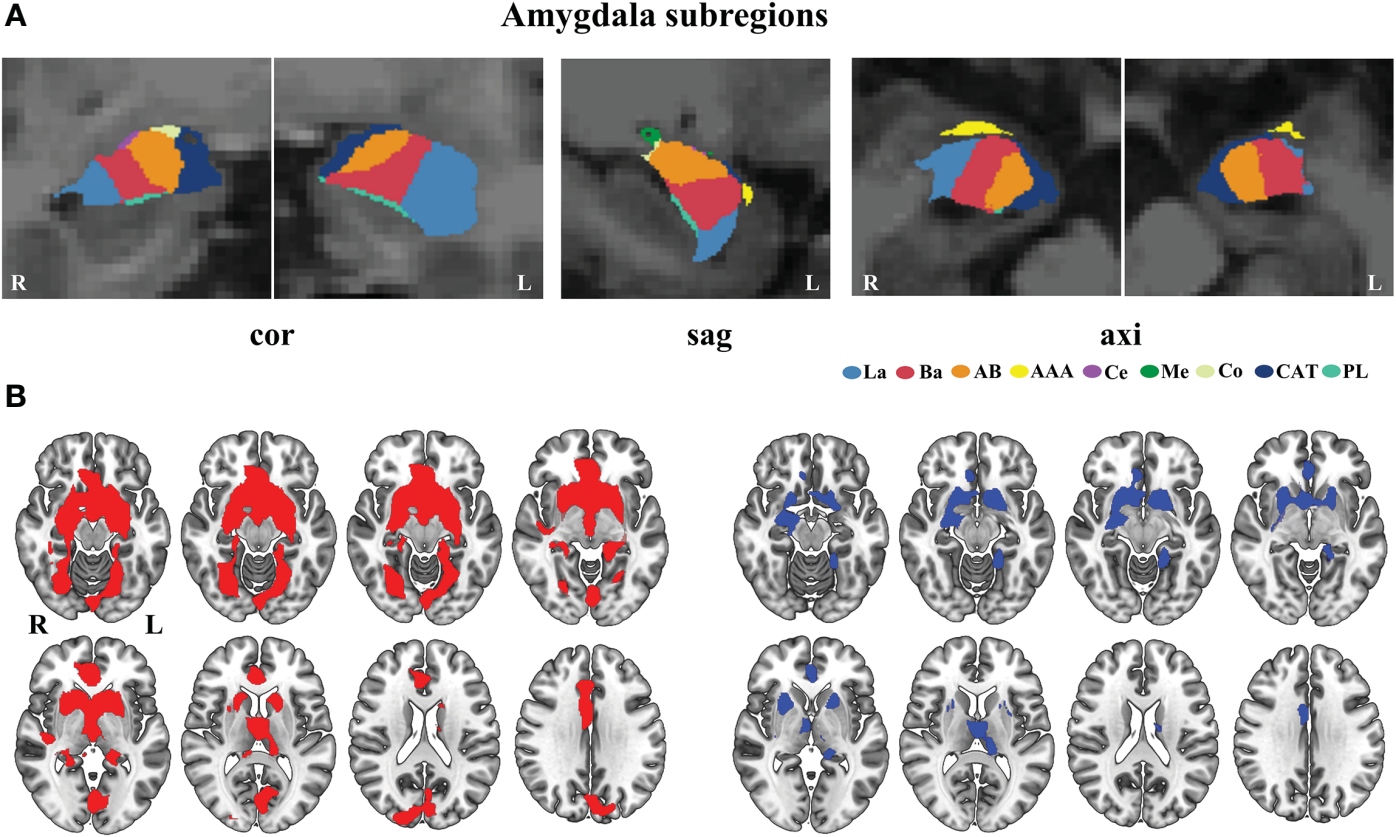
Comparison of the altered GMV covariance between the amygdala subregions and the whole amygdala. **(A)** Visualization of amygdala subregions in the brain. **(B)** The different mask of the significant GMV covariance results for the amygdala subregions (red color) and whole amygdala (blue color). axi, axial, cor, coronal, sag, sagittal. AAA, anterior-amygdaloid-area; AB, accessory-basal nucleus; Ba, basal nucleus; CAT, corticoamygdaloid-transition; Ce, central nucleus; Co, cortical nucleus; La, lateral nucleus; Me, medial nucleus; PL, paralaminar nucleus; L, left; R, right.

In **Figure 2A**, we present a visualization of the amygdala subregions. In **Figure 2B**, we demonstrated two binary masks for amygdala subregions versus whole amygdala, allowing us to compare significant findings between amygdala subregions and the whole amygdala. We observed that the whole amygdala only showed increased GMV covariance with the bilateral thalamus and basal ganglia. In contrast, the amygdala subregion analyses revealed more distributed brain regions with increased covariance, including the frontal, occipital, and temporal lobes. Our results demonstrated that the subregional analysis could uncover more GMV covariance abnormalities and may improve our understanding of the different roles of amygdala subregions in the pathological process of schizophrenia.

**Figure 3:**
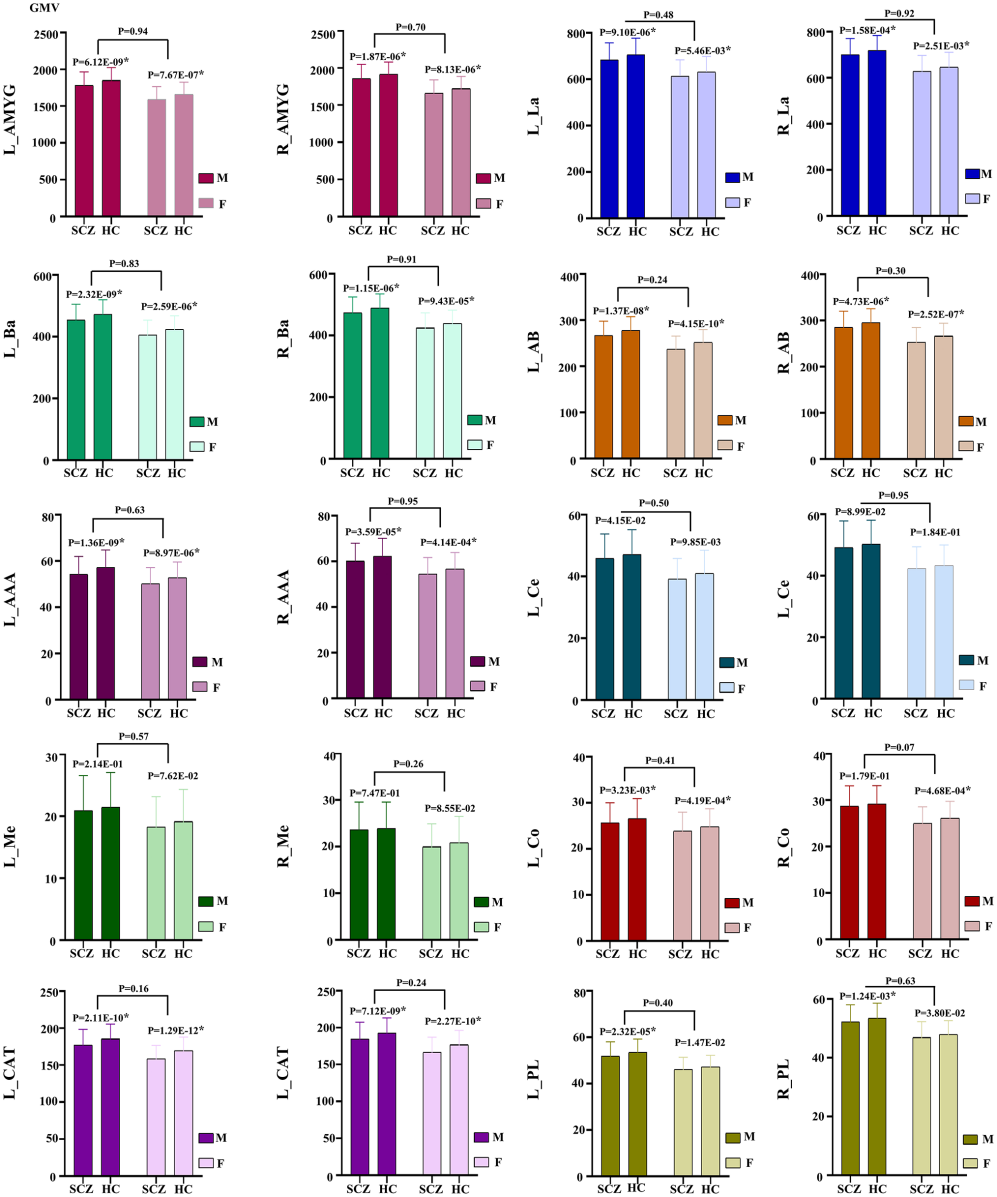
GMV alteration of the amygdala and its subregions in schizophrenia patients between the male and female subgroups. * indicates corrected by multiple comparisons. AAA, anterior-amygdaloid-area; AB, accessory-basal nucleus; Ba, basal nucleus; CAT, corticoamygdaloid-transition; Ce, central nucleus; Co, cortical nucleus; La, lateral nucleus; Me, medial nucleus; PL, paralaminar nucleus; L, left; R, right; SCZ, Schizophrenia; HC, Healthy control.

At the same time, our analysis revealed that the GMV was significantly reduced in the whole amygdala across both hemispheres for both male and female subgroups of schizophrenia patients. In the male subgroup, GMV was significantly lower in all subregions except for bilateral Ce, Me and right Co. Conversely, in the female subgroup, all subregions exhibited significantly reduced GMV except for the bilateral Ce, Me, PL. There were no inter-sex differences in the observed differences between the male and female groups (*P* > 0.05) (**Figure 3**).

### Specific differential GMV covariance patterns of amygdala subregions

According to the target ROIs defined in the methods, we selected 28 target ROIs, including pregenual part of anterior cingulate cortex (ACCpre), subgenual part of anterior cingulate cortex (ACCsub), supra callosal part of anterior cingulate cortex (ACCsup), caudate nucleus (CAU), hippocampus (HIP), accumbens nucleus (Nacc), olfactory cortex (OLF), parahippocampal gyrus (PHG), putamen (PUT), pallidum (PAL), thalamus (THA) in the bilateral cerebral hemispheres, medial orbital part of superior frontal gyrus (PFCmed), and fusiform gyrus (FFG) in the right cerebral hemisphere, and calcarine fissure and surrounding cortex (CAL), cuneus (CUN), lingual gyrus (LING), and rectus gyrus (REC) in the left cerebral hemisphere.

**Figure 4:**
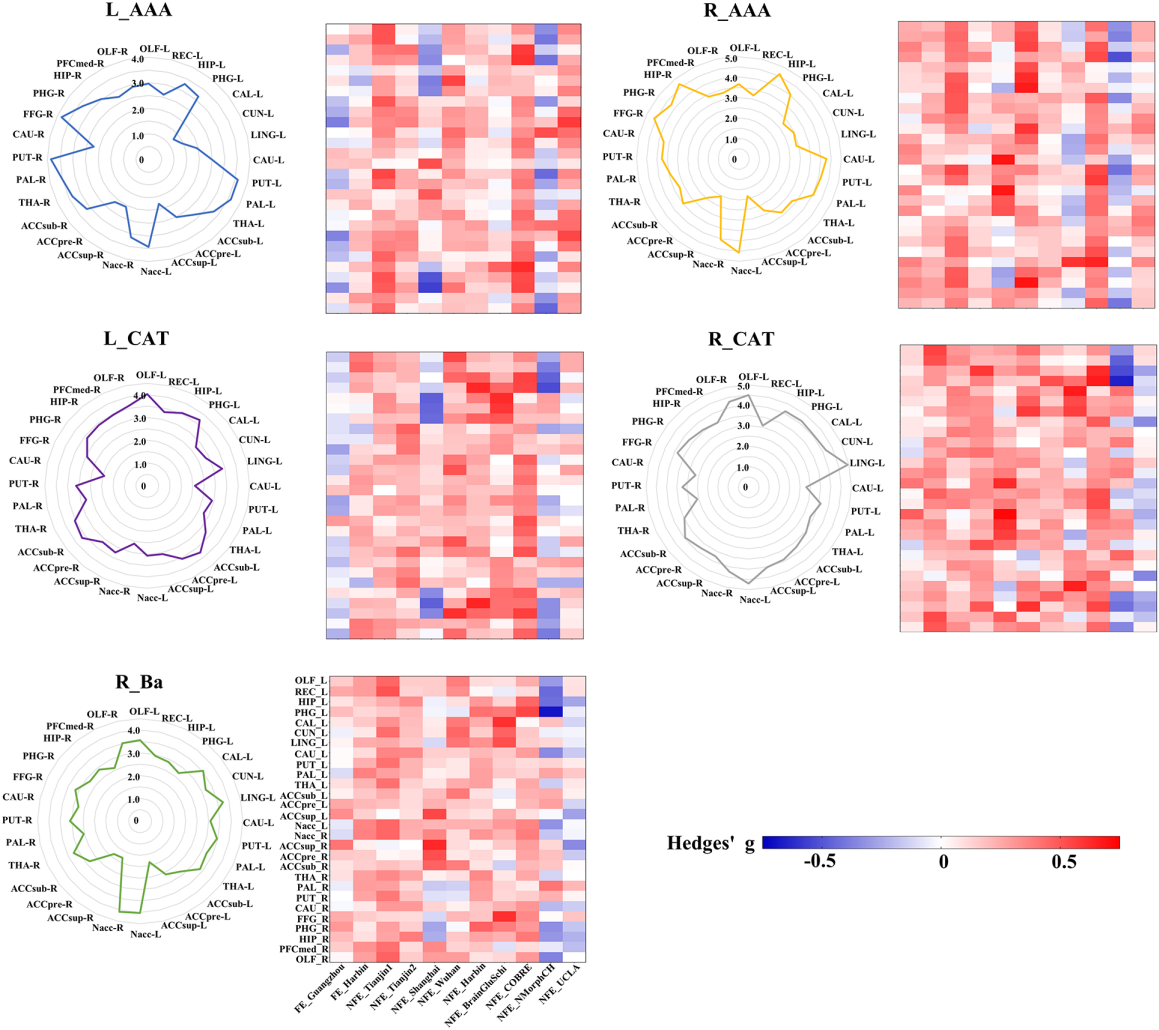
GMV covariance fingerprints of the five amygdala subregions and the heat map exhibiting validation across centers. The scale value of the fingerprint graph represents the *t*-value of inter-group differences in GMV covariance. The colorbar of the heat map represents the Hedges’ adjusted g* of the inter-group GMV covariance fingerprints of the five amygdala subregions in each centers. ACCpre, pregenual part of anterior cingulate cortex; ACCsub, subgenual part of anterior cingulate cortex; ACCsup, supra callosal part of anterior cingulate cortex; CAL, calcarine fissure and surrounding cortex; CAU, caudate nucleus; CUN, cuneus; FFG, fusiform gyrus; HIP, hippocampus; LING, lingual gyrus; Nacc, accumbens nucleus; OFCmed, medial orbital gyrus; OLF, olfactory cortex; PAL, pallidum; PHG, parahippocampal gyrus; PUT, putamen; REC, rectus gyrus; THA, thalamus. L_AAA, Anterior-amygdaloid-area of left hemisphere; R_AAA, Anterior-amygdaloid-area of right hemisphere; L_CAT, Corticoamygdaloid-transitio of left hemisphere; R_CAT, Corticoamygdaloid-transitio of right hemisphere; R_Ba, Basal nucleus of right hemisphere.

To visualize the specific differential GMV covariance patterns of each amygdala subregion, we calculated the differential GMV covariance fingerprints between each amygdala subregion and the 28 target ROIs (**Figure 4**). Relative to the HC, the L_AAA of schizophrenia exhibited mostly strengthened covariance with bilateral PUT; R_AAA had stronger covariance with bilateral HIP in schizophrenia; L_CAT mainly showed greater covariance with the OLF_L in schizophrenia; R_CAT had higher covariance with the LING_L and Nacc_L in schizophrenia; R_Ba showed stronger covariance with the bilateral Nacc in schizophrenia. In the validation of subregional GMV covariance specificity between different centers, we found that our results were consistent in most centers to an extent (**Figure 4**). Specifically, the covariance pattern of L_AAA was not stable in FE_Guangzhou, and the non-first-episode centers could be verified except for Shanghai and NMorphCH. The covariance pattern of R_AAA could be validated except for NFE_BrainGluSchi and NFE_NMorphCH. The findings of L_CAT could not be validated in the FE_Guangzhou, NFE_Shanghai, and NFE_NMorphCH centers. The GMV covariance patterns of R_CAT and R_Ba were not replicated in the NFE_NMorphCH and NFE_UCLA centers.

**Figure 5:**
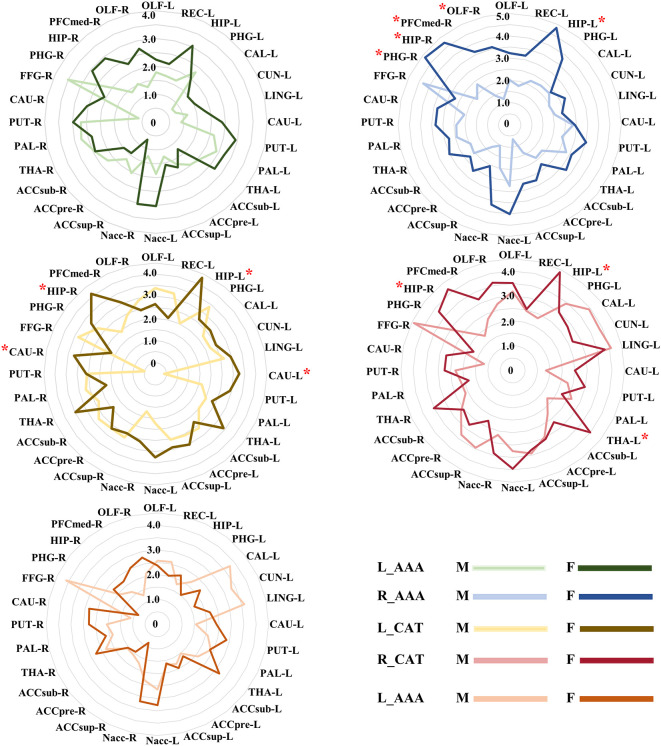
The different GMV covariance fingerprints for five amygdala subregions in male and female subjects. * indicates trends in sex differences, uncorrected. ACCpre, pregenual part of anterior cingulate cortex; ACCsub, subgenual part of anterior cingulate cortex; ACCsup, supra callosal part of anterior cingulate cortex; CAL, calcarine fissure and surrounding cortex; CAU, caudate nucleus; CUN, cuneus; FFG, fusiform gyrus; HIP, hippocampus; LING, lingual gyrus; Nacc, accumbens nucleus; OFCmed, medial orbital gyrus; OLF, olfactory cortex; PAL, pallidum; PHG, parahippocampal gyrus; PUT, putamen; REC, rectus gyrus; THA, thalamus. L_AAA, Anterior-amygdaloid-area of left hemisphere; R_AAA, Anterior-amygdaloid-area of right hemisphere; L_CAT, Corticoamygdaloid-transitio of left hemisphere; R_CAT, Corticoamygdaloid-transitio of right hemisphere; R_Ba, Basal nucleus of right hemisphere. M, male, F, female.

In our sex-specific analysis experiment, we found that GMV covariance fingerprints exhibited distinct patterns across sexes, with these patterns being more pronounced in women. Specifically, in R_AAA, there was an observed trend towards sex differences in HIP_L, PHG_R, HIP_R, PFCmed_R and OLF_R (*P*< 0.05, uncorrected). In L_CAT, we identified sex-differentiated trends in bilateral HIP and CAU (*P*< 0.05, uncorrected). Similarly, in R_CAT, there was a trend towards sex differences in bilateral HIP and left THA (P< 0.05, uncorrected) (**Figure 5**).

### Association between the GMV covariance and clinical information

A Spearman correlation analysis was performed between the GMV covariance alteration and the clinical information in schizophrenia patients. There was a weak positive association between the positive syndrome scale total score and the GMV covariance of R_AAA & CUN_L (*P* = 0.034, uncorrected), R_AAA & PAL_L (*P* = 0.026, uncorrected), R_AAA & Nacc_L (*P* = 0.021, uncorrected). The total score of negative syndrome scale showed a negative association with the GMV covariance of L_CAT & CAU_L (*P* = 0.047), L_CAT & PAL_R (*P* = 0.038), L_CAT & CAU_R (*P* = 0.023), R_CAT & ACCsup_R (*P* = 0.046), R_Ba & CAU_L (*P* = 0.016), R_Ba & CAU_R (*P* = 0.017). There was a weak positive association between general psychopathology scale total score with the GMV covariance of L_AAA & OLF_L (*P* = 0.022, uncorrected), L_AAA & REC_L (*P* = 0.047, uncorrected), L_AAA & Nacc_L (*P* = 0.041, uncorrected), L_AAA & PFCmed_R (*P* = 0.049, uncorrected), L_CAT & Nacc_L (*P* = 0.049, uncorrected), R_AAA & OLF_L (*P* = 0.020, uncorrected), R_AAA & CUN_L (*P* = 0.014, uncorrected), R_AAA & PHG_R (*P* = 0.046, uncorrected), R_AAA & OLF_R (*P* = 0.032, uncorrected), R_CAT & Nacc_L (*P* = 0.037, uncorrected), R_CAT & Nacc_R (*P* = 0.048, uncorrected), R_CAT & FFG_R (*P* = 0.036, uncorrected), R_Ba & CUN_L (*P* = 0.034, uncorrected), R_Ba & Nacc_L (*P* = 0.048, uncorrected). We identified no association between PANSS total score and amygdala GMV covariance patterns. We revealed a weak negative correlation between disease course information and GMV covariance of L_AAA & ACCsup_R (*P* = 0.39, uncorrected), L_CAT & ACCsup_R (*P* = 0.045, uncorrected), R_AAA & THA_L (*P* = 0.038, uncorrected), R_CAT & ACCsup_R (*P* = 0.036, uncorrected), R_Ba & ACCsup_R (*P* = 0.018, uncorrected). Finally, we revealed an uncorrected negative association between the CPZ with the GMV covariance of L_AAA & CUN_L (*P* = 0.030), L_AAA & ACCsub_L (*P* = 0.006), L_AAA & ACCsub_R (*P* = 0.007), L_CAT & ACCsub_L (*P* = 0.031), L_CAT & ACCpre_R (*P* = 0.037), L_CAT & ACCsub_R (*P* = 0.011), R_AAA & PAL_L (*P* = 0.006), R_AAA & ACCsub_R (*P* = 0.018), R_AAA & PAL_R (*P* = 0.005), R_AAA & FFG_R (*P* = 0.019), R_CAT & CUN_L (*P* = 0.008), R_CAT & PHG_R (*P* = 0.048), R_Ba & HIP_L (*P* = 0.043), R_Ba & PHG_L (*P* = 0.011), R_Ba & PHG_R (*P* = 0.014), R_Ba & HIP_R (*P* = 0.016).

## Discussion

To our knowledge, this is the first study to investigate the GMV covariance changes of the amygdala in schizophrenia at the subregion level. We used extensive data from multicenters and verified that only 5/18 amygdala subregions in schizophrenia patients showed replicable increased GMV covariance compared to healthy controls. Moreover, strengthened GMV covariance patterns are relatively unique for each of the five amygdala subregions in individuals with schizophrenia. These findings support our hypothesis that the GMV covariances of amygdala subregions are selectively impaired in schizophrenia.

Combining structural brain MRI data from 807 patients with schizophrenia and 845 healthy controls from 11 centers, we calculated the robustness of effect sizes and assessed variability across centers. Most remarkably, our study found extensive GMV covariance enhancement between five subregions of the amygdala and the frontal, occipital, and temporal cortex and in SCZ patients, which is consistent with the hypothesis that SCZ is a widespread dysconnection disorder (8, 49, 50). The enhanced GMV covariance in SCZ patients may indicate coordinated GMV loss throughout neurodevelopment (51, 52). In fact, schizophrenia patients also exhibits widespread atrophy in amygdala, frontal, occipital, temporal cortex, and basal ganglia, which may be caused by excessive synaptic pruning during adolescence (53, 54). We speculated that the GMV covariance perturbations between amygdala subregions and other brain areas may result from shared common causal factors. Future studies are expected to focus on the effects of genetic and environmental factors on the impairment of amygdala subregions’ structure and their covariance, which may provide new perspectives to unravel the etiology and mechanisms of amygdala damage in schizophrenia (55, 56).

Previous postmortem investigations in schizophrenia patients reported reduced mean total neuron number in the lateral nucleus of the amygdala (31), as well as alterations in the nuclear area, nucleolar volume (29), and oligodendrocyte density (57) in the basolateral complex. Recent *in vivo* studies have shown that all amygdala nuclei have reduced in size (30) except the medial nucleus, and primarily impaired amygdala nuclei are the basal and lateral nuclei (22) or the right basolateral complex (58), supporting our findings with regional atrophy of amygdala subfields.

Moreover, we found significantly increased GMV covariance patterns in five amygdala subregions in schizophrenia patients, including the right basal nucleus, bilateral anterior-amygdaloid-area, and bilateral bilateral corticoamygdaloid-transition. The basal nucleus, a part of the basolateral complex, undergoes marked atrophy in schizophrenia (59). The basal nucleus receives input from the lateral nucleus and transmits signals to the central nucleus, which is involved in the expression and extinction of fear (60). And has extensive connections with ventral striatal areas and the orbital prefrontal cortex (61), which are important in integrating, coordinating, and processing of external sensory input (27, 62). It has been demonstrated that the basal amygdala has strong functional connectivity with the regions of the ventral caudal, medial frontal, and caudal orbitofrontal cortex. Functional connectivity between the accumbens nucleus (Nacc) and the basal amygdala is positively associated with negative emotions (6, 63). Simultaneously, we observed a significant enhanced GMV covariance pattern between the right basal nucleus and Nacc/OLF. Thus, the GMV covariance abnormalities in the basolateral complex (consisting of the basal, lateral, and accessory basal nuclei) may be linked to dysfunctional emotional regulation and ensuing deficiencies in adaptive behavior in schizophrenia.

We also found the corticoamygdaloid-transition area (CAT) had increased GMV covariance with the hippocampus, parahippocampal gyrus, olfactory cortex, and anterior cingulate cortex. The hippocampus, as one of the prominently affected brain areas in the pathogenesis of schizophrenia, is a core region for learning and episodic memory (64). There is evidence that CAT receives inputs from the hippocampus and outputs the emotional context of memories to inferior neurons (63, 65). Hence, disrupting connections between the amygdala and hippocampus may affect memories of emotionally relevant events in schizophrenia (29, 66). Additional studies have demonstrated a negative correlation between CAT volume and salivary cortisol (67). Stress is one of the main factors contributing to the production of cortisol, and this stress exposure greatly increases the risk of schizophrenia (68, 69). Preliminary evidence suggests that the CAT is involved in assessing negative emotions (70). Deficits in facial emotion interpretation and social skills in schizophrenic patients may stem from a reduction in the volume of the CAT (30). Our finding provides additional evidence for its involvement in emotional processing. Finally, athough there is evidence of a significant reduction in GMV of AAA in schizophrenia patients (30), it is difficult to draw conclusions regarding the involvement of AAA in schizophrenia since little is known about its connections and functions.

We further conducted a sex-specific analysis and found that there were sex differences in GMV covariance fingerprints, the female was more pronounced. However, the sample size of the male and female groups was different. The sex differences in GMV covariance in schizophrenia patients can be attributed to several biological, genetic, and hormonal factors that interact with the disease’s pathophysiology (71–73). Understanding these differences was crucial for developing sex-specific approaches to treatment and management, which needs further exploration.

A large number of previous studies have shown varying degrees of correlation between reduced GMV in brain regions and negative or positive symptoms in patients with schizophrenia (57, 74). However, we did not obtain significant results in our study on the correlation between the altered GMV covariance and clinical symptoms, consistent with the findings of Spreng et al (75). Investigating the lack of correlation between GMV covariance alterations and the clinical features in schizophrenia patients offers a nuanced view into the complexities of the disease. This phenomenon can be attributed to several factors: 1) Schizophrenia is highly heterogeneous (2), with significant variability in pathology and progression among individuals. This heterogeneity might obscure any consistent correlation between GMV covariance alterations and clinical features across a broad sample of patients; 2) Different stages of schizophrenia (e.g., early vs. late) may impact brain structure in varying ways, and the progression rate can differ among individuals. Clinical features capture symptoms at a specific point in time, whereas GMV covariance alterations might reflect the cumulative effects of the disease, making synchronous correlation unlikely (20, 76). We propose that our findings, while preliminary, have the potential to be an effective imaging marker for differentiating schizophrenia from healthy populations and open avenues for future research. However, further studies are still needed to validate its differentiation value. We validated the GMV covariance model separately for the data between each center, and consistent results were obtained at most centers. Validation was not obtained from all centers, and we argued that the possible reasons included inconsistency in sample size between centers, differences in MRI models and scanning parameters between centers, and diversity in the causes of patient morbidity (77–79).

There are some limitations in our study. First, due to a lack of information on the PANSS, illness history, and antipsychotic medication usage, it was not feasible to determine if the altered GMV covariance patterns were correlated with the existence of clinical measures. Second, the etiology and biological pathways of GMV covariation in the amygdala subregion of schizophrenia are still unknown.

In conclusion, our study discovered a selective disruption of GMV covariance of amygdala subregions in a large sample of schizophrenia patients for the first time. We validated the reproducibility of our findings in 11 centers containing first-episode and non-first-episode schizophrenia patients. Further study is needed to disentangle the biological mechanisms and clinical significance of perturbed GMV covariance of amygdala subregions.

## Data availability statement

The original contributions presented in the study are included in the article/**Supplementary Material**, further inquiries can be directed to the corresponding author.

## Ethics statement

The studies involving humans were approved by the Ethics Committee of Tianjin Medical University General Hospital, Tianjin, China. The patients/participants provided their written informed consent to participate in this study. Relevant Institutional Review Boards also approved the four public test-retest datasets, and detailed recruitment information was provided on the website. The studies were conducted in accordance with the local legislation and institutional requirements. Written informed consent for participation was not required from the participants or the participants’ legal guardians/next of kin in accordance with the national legislation and institutional requirements.

## Author contributions

ZC: Formal analysis, Visualization, Writing – original draft, Writing – review & editing. LL: Data curation, Writing – original draft. LL: Data curation, Writing – original draft. GW: Data curation, Writing – original draft. CZ: Data curation, Writing – original draft. HT: Data curation, Writing – original draft. WL: Data curation, Writing – original draft. LW: Data curation, Writing – original draft. BZ: Data curation, Writing – original draft. JR: Data curation, Writing – original draft. YZ: Formal analysis, Data curation, Writing – original draft. YX: Data curation, Writing – original draft. XD: Data curation, Writing – original draft. XW: Data curation, Writing – original draft. LW: Data curation, Writing – original draft. YL: Data curation, Writing – original draft. HD: Data curation, Writing – original draft. XL: Data curation, Writing – original draft. ZZ: Data curation, Writing – original draft. ML: Funding acquisition, Writing – original draft. CZ: Data curation, Writing – original draft. XW: Data curation, Writing – review & editing. CY: Funding acquisition, Writing – review & editing. WQ: Methodology, Funding acquisition, Writing – review & editing. HL: Methodology, Funding acquisition, Writing – review & editing.
