# Supplementary material for: Selective disrupted gray matter volume covariance of amygdala subregions in schizophrenia
Source: Front Psychiatry. 2024 Apr 29;15:1349989. doi: 10.3389/fpsyt.2024.1349989 (PMC11090100; doi:10.3389/fpsyt.2024.1349989)
Supplement: Supplementary file 1 [file DataSheet_1.pdf]

Supplementary Materials

Supplementary Table 1. Spearman correlation analysis of amygdala subregion covariance with positive scale total score

	L_AAA		L_CAT		R_AAA		R_CAT		R_Ba	
	r	*P*	*r*	*P*	*r*	*P*	*r*	*P*	*r*	*P*
OLF_L	0.041	0.332	0.043	0.306	0.067	0.112	0.023	0.589	0.026	0.544
REC_L	0.043	0.305	0.047	0.267	0.055	0.191	0.000	0.998	0.008	0.855
HIP_L	0.042	0.317	0.020	0.643	0.040	0.343	0.032	0.455	0.035	0.404
PHG_L	0.036	0.394	0.025	0.563	0.075	0.077	0.048	0.258	0.052	0.221
CAL_L	0.024	0.564	-0.010	0.814	0.052	0.215	0.022	0.598	0.038	0.373
CUN_L	0.061	0.147	0.020	0.645	**0.089**	**0.034***	0.030	0.474	0.036	0.398
LING_L	0.036	0.398	0.005	0.911	0.056	0.182	-0.013	0.754	0.011	0.797
CAU_L	0.051	0.231	0.066	0.116	0.056	0.186	0.053	0.208	0.037	0.386
PUT_L	0.031	0.461	0.038	0.372	0.067	0.112	0.051	0.228	0.069	0.101
PAL_L	0.045	0.286	0.047	0.264	**0.094**	**0.026***	0.044	0.299	0.079	0.060
THA_L	0.029	0.495	-0.004	0.935	0.026	0.539	0.025	0.552	0.025	0.562
ACCsub_L	-0.009	0.825	0.012	0.783	0.008	0.847	-0.021	0.620	-0.004	0.928
ACCpre_L	-0.020	0.643	-0.012	0.781	-0.020	0.639	-0.039	0.362	-0.021	0.628
ACCsup_L	0.001	0.983	-0.002	0.972	0.015	0.727	-0.041	0.330	0.000	0.999
Nacc_L	0.064	0.132	0.080	0.058	**0.097**	**0.021***	0.062	0.140	0.077	0.070
Nacc_R	0.009	0.837	0.050	0.237	0.065	0.122	0.052	0.221	0.057	0.179
ACCsup_R	0.037	0.380	0.043	0.313	0.020	0.633	0.005	0.899	0.020	0.640
ACCpre_R	0.042	0.321	0.042	0.320	0.027	0.530	-0.008	0.857	0.010	0.821
ACCsub_R	-0.004	0.934	0.023	0.581	0.004	0.924	-0.025	0.551	-0.023	0.583
THA_R	0.048	0.255	0.041	0.336	0.026	0.541	0.032	0.455	0.021	0.618
PAL_R	0.010	0.815	0.022	0.600	0.057	0.178	0.052	0.218	0.068	0.106

PUT_R	0.038	0.371	0.044	0.297	0.068	0.109	0.058	0.168	0.070	0.095
CAU_R	0.026	0.546	0.069	0.105	0.039	0.361	0.052	0.218	0.026	0.542
FFG_R	0.036	0.401	0.019	0.649	0.064	0.127	0.029	0.493	0.047	0.265
PHG_R	0.004	0.922	0.023	0.594	0.062	0.141	0.034	0.420	0.034	0.424
HIP_R	0.015	0.716	0.005	0.902	0.037	0.383	0.013	0.751	0.024	0.574
PFCmed_R	0.031	0.458	0.040	0.349	0.039	0.354	0.003	0.943	0.011	0.797
OLF_R	0.010	0.820	0.035	0.410	0.055	0.193	0.024	0.563	0.025	0.549

* Significant results, $P < 0.05$, Uncorrected.

Supplementary Table 2. Spearman correlation analysis of amygdala subregion covariance with negative scale total score

	L_AAA		L_CAT		R_AAA		R_CAT		R_Ba	
	r	*P*	*r*	*P*	*r*	*P*	*r*	*P*	*r*	*P*
OLF_L	0.011	0.803	-0.043	0.305	-0.007	0.864	-0.042	0.320	-0.029	0.492
REC_L	-0.015	0.717	-0.049	0.247	-0.037	0.388	-0.056	0.184	-0.051	0.231
HIP_L	-0.023	0.590	-0.056	0.189	-0.036	0.396	-0.044	0.299	-0.033	0.440
PHG_L	0.003	0.950	-0.020	0.640	0.004	0.919	0.001	0.980	-0.026	0.546
CAL_L	-0.021	0.620	-0.034	0.427	-0.026	0.544	-0.049	0.248	-0.056	0.189
CUN_L	0.031	0.466	-0.032	0.454	0.004	0.924	-0.046	0.280	-0.022	0.605
LING_L	0.002	0.956	-0.030	0.477	-0.009	0.831	-0.047	0.268	-0.066	0.117
CAU_L	-0.040	0.351	**-0.084**	**0.047***	-0.066	0.117	-0.063	0.138	**-0.101**	**0.016***
PUT_L	-0.004	0.921	-0.055	0.197	-0.022	0.600	-0.035	0.405	-0.029	0.495
PAL_L	-0.024	0.572	-0.082	0.052	-0.018	0.679	-0.061	0.147	-0.040	0.348
THA_L	-0.014	0.734	-0.038	0.374	-0.026	0.541	-0.046	0.272	-0.050	0.233
ACCsub_L	-0.018	0.676	0.002	0.961	-0.026	0.533	-0.035	0.403	-0.028	0.505
ACCpre_L	-0.046	0.277	-0.030	0.482	-0.062	0.143	-0.046	0.277	-0.036	0.392
ACCsup_L	-0.052	0.222	-0.031	0.469	-0.042	0.321	-0.068	0.109	-0.050	0.239
Nacc_L	0.013	0.758	-0.030	0.477	-0.015	0.718	-0.028	0.515	-0.021	0.618
Nacc_R	-0.009	0.831	-0.047	0.262	-0.044	0.294	-0.032	0.447	-0.035	0.408
ACCsup_R	-0.056	0.189	-0.033	0.431	-0.063	0.137	**-0.084**	**0.046***	-0.056	0.186
ACCpre_R	-0.004	0.933	-0.035	0.410	-0.045	0.286	-0.064	0.132	-0.030	0.478
ACCsub_R	0.006	0.897	-0.002	0.965	-0.018	0.663	-0.041	0.336	-0.037	0.386
THA_R	-0.029	0.489	-0.011	0.797	-0.047	0.265	-0.015	0.725	-0.029	0.499
PAL_R	-0.033	0.433	**-0.088**	**0.038***	-0.034	0.427	-0.055	0.189	-0.035	0.405
PUT_R	0.002	0.962	-0.055	0.196	-0.021	0.615	-0.033	0.437	-0.021	0.627
CAU_R	-0.035	0.412	**-0.096**	**0.023***	-0.063	0.135	-0.074	0.081	**-0.101**	**0.017***

FFG_R	-0.001	0.980	-0.041	0.331	-0.026	0.538	-0.005	0.901	-0.037	0.384
PHG_R	0.018	0.675	-0.033	0.436	0.023	0.589	-0.012	0.780	-0.037	0.384
HIP_R	-0.007	0.863	-0.063	0.138	-0.022	0.612	-0.049	0.242	-0.039	0.363
PFCmed_R	0.009	0.825	-0.026	0.538	-0.021	0.623	-0.020	0.643	-0.027	0.530
OLF_R	0.019	0.655	-0.058	0.173	-0.016	0.710	-0.048	0.255	-0.030	0.472

* Significant results, $P < 0.05$, Uncorrected.

Supplementary Table 3. Spearman correlation analysis of amygdala subregion covariance with general psychopathology scale total score

	L_AAA		L_CAT		R_AAA		R_CAT		R_Ba	
	r	*P*	*r*	*P*	*r*	*P*	*r*	*P*	*r*	*P*
OLF_L	**0.097**	**0.022***	0.069	0.103	**0.098**	**0.020***	0.071	0.091	0.075	0.078
REC_L	**0.084**	**0.047***	0.066	0.121	0.076	0.071	0.048	0.258	0.057	0.181
HIP_L	0.036	0.390	0.017	0.681	0.038	0.365	0.033	0.437	0.024	0.577
PHG_L	0.063	0.134	0.043	0.314	0.078	0.067	0.079	0.061	0.042	0.323
CAL_L	0.047	0.262	0.008	0.843	0.066	0.120	0.062	0.143	0.078	0.065
CUN_L	0.083	0.050	0.023	0.587	**0.103**	**0.014***	0.054	0.205	**0.090**	**0.034***
LING_L	0.067	0.113	0.039	0.359	0.061	0.147	0.045	0.292	0.049	0.250
CAU_L	-0.002	0.972	0.007	0.870	-0.015	0.731	0.021	0.622	-0.014	0.735
PUT_L	0.024	0.566	0.028	0.511	0.056	0.183	0.048	0.256	0.057	0.177
PAL_L	0.037	0.380	0.023	0.593	0.081	0.056	0.035	0.414	0.059	0.165
THA_L	0.028	0.512	-0.008	0.845	0.011	0.793	0.000	0.992	0.005	0.905
ACCsub_L	0.030	0.472	0.044	0.298	0.041	0.333	0.035	0.413	0.034	0.418
ACCpre_L	0.011	0.787	0.017	0.680	0.015	0.732	0.017	0.684	0.025	0.557
ACCsup_L	0.007	0.864	0.016	0.703	0.019	0.654	-0.011	0.792	0.013	0.762
Nacc_L	**0.086**	**0.041***	**0.083**	**0.049***	0.082	0.052	**0.088**	**0.037***	**0.083**	**0.048***
Nacc_R	0.031	0.459	0.051	0.229	0.048	0.261	**0.084**	**0.048***	0.070	0.098
ACCsup_R	0.011	0.799	0.040	0.339	0.006	0.889	-0.012	0.771	0.016	0.698
ACCpre_R	0.063	0.137	0.044	0.298	0.044	0.300	0.018	0.663	0.049	0.245
ACCsub_R	0.048	0.255	0.041	0.338	0.043	0.307	0.025	0.561	0.025	0.557
THA_R	0.048	0.259	0.036	0.392	0.021	0.625	0.037	0.376	0.027	0.517
PAL_R	0.016	0.705	0.004	0.919	0.056	0.183	0.038	0.368	0.055	0.195
PUT_R	0.038	0.367	0.036	0.392	0.064	0.130	0.056	0.187	0.069	0.101
CAU_R	-0.011	0.788	0.001	0.973	-0.015	0.721	0.017	0.695	-0.018	0.676

FFG_R	0.079	0.062	0.025	0.560	0.058	0.171	**0.089**	**0.036***	0.069	0.103
PHG_R	0.047	0.262	0.040	0.346	**0.084**	**0.046***	0.062	0.140	0.026	0.543
HIP_R	0.025	0.553	-0.002	0.970	0.047	0.263	0.019	0.658	0.016	0.699
PFCmed_R	**0.083**	**0.049***	0.069	0.103	0.082	0.053	0.082	0.052	0.067	0.115
OLF_R	0.076	0.072	0.048	0.260	**0.091**	**0.032***	0.066	0.117	0.080	0.058

* Significant results, $P < 0.05$, Uncorrected.

Supplementary Table 4. Spearman correlation analysis of amygdala subregion covariance with PANSS total score

	L_AAA		L_CAT		R_AAA		R_CAT		R_Ba	
	r	*P*	*r*	*P*	*r*	*P*	*r*	*P*	*r*	*P*
OLF_L	0.063	0.139	0.027	0.528	0.064	0.129	0.023	0.589	0.031	0.462
REC_L	0.052	0.221	0.026	0.545	0.043	0.305	0.001	0.978	0.012	0.770
HIP_L	0.019	0.656	-0.010	0.818	0.014	0.748	0.012	0.776	0.008	0.847
PHG_L	0.041	0.332	0.020	0.643	0.060	0.155	0.058	0.168	0.026	0.547
CAL_L	0.020	0.629	-0.012	0.776	0.034	0.420	0.021	0.615	0.028	0.514
CUN_L	0.069	0.104	0.004	0.927	0.078	0.063	0.019	0.659	0.046	0.281
LING_L	0.044	0.295	0.010	0.817	0.041	0.337	0.003	0.945	0.001	0.979
CAU_L	0.000	0.997	-0.003	0.946	-0.014	0.733	0.008	0.848	-0.029	0.500
PUT_L	0.019	0.653	0.004	0.924	0.037	0.376	0.024	0.571	0.036	0.400
PAL_L	0.022	0.598	-0.006	0.884	0.061	0.152	0.008	0.851	0.038	0.368
THA_L	0.014	0.740	-0.023	0.583	0.002	0.960	-0.008	0.842	-0.010	0.821
ACCsub_L	0.004	0.925	0.020	0.644	0.010	0.809	-0.006	0.892	0.002	0.962
ACCpre_L	-0.018	0.679	-0.011	0.798	-0.023	0.581	-0.024	0.577	-0.011	0.801
ACCsup_L	-0.016	0.703	-0.009	0.834	-0.005	0.899	-0.046	0.275	-0.017	0.695
Nacc_L	0.063	0.136	0.052	0.220	0.062	0.144	0.050	0.239	0.054	0.198
Nacc_R	0.013	0.752	0.023	0.582	0.026	0.539	0.046	0.280	0.039	0.354
ACCsup_R	-0.007	0.865	0.015	0.717	-0.021	0.618	-0.038	0.375	-0.011	0.803
ACCpre_R	0.042	0.319	0.018	0.671	0.012	0.786	-0.019	0.648	0.013	0.758
ACCsub_R	0.022	0.601	0.020	0.642	0.013	0.763	-0.014	0.740	-0.012	0.783
THA_R	0.023	0.586	0.022	0.599	0.000	0.999	0.021	0.616	0.008	0.859
PAL_R	-0.001	0.986	-0.023	0.580	0.030	0.474	0.016	0.707	0.035	0.407
PUT_R	0.032	0.453	0.011	0.802	0.042	0.325	0.033	0.441	0.046	0.281
CAU_R	-0.011	0.788	-0.008	0.848	-0.021	0.624	0.001	0.983	-0.035	0.412

FFG_R	0.050	0.239	0.005	0.905	0.034	0.421	0.052	0.219	0.032	0.447
PHG_R	0.031	0.458	0.015	0.716	0.065	0.123	0.040	0.344	0.008	0.852
HIP_R	0.014	0.748	-0.024	0.575	0.023	0.591	-0.003	0.936	-0.002	0.965
PFCmed_R	0.054	0.204	0.033	0.439	0.044	0.298	0.032	0.446	0.025	0.559
OLF_R	0.045	0.284	0.008	0.850	0.054	0.203	0.020	0.645	0.034	0.418

* Significant results, $P < 0.05$, Uncorrected.

Supplementary Table 5. Spearman correlation analysis of amygdala subregion covariance with course of disease

	L_AAA		L_CAT		R_AAA		R_CAT		R_Ba	
	r	*P*	*r*	*P*	*r*	*P*	*r*	*P*	*r*	*P*
OLF_L	0.012	0.776	0.007	0.857	0.001	0.980	0.013	0.758	-0.014	0.735
REC_L	0.031	0.456	0.016	0.707	0.039	0.340	0.050	0.224	0.011	0.794
HIP_L	0.013	0.751	0.000	0.999	-0.043	0.294	0.012	0.767	-0.039	0.340
PHG_L	0.000	0.996	0.016	0.700	-0.013	0.745	0.034	0.409	0.001	0.985
CAL_L	0.002	0.953	-0.033	0.428	-0.034	0.412	-0.026	0.521	-0.061	0.140
CUN_L	-0.033	0.427	-0.058	0.155	-0.020	0.621	-0.017	0.687	-0.035	0.389
LING_L	0.018	0.655	-0.011	0.782	-0.014	0.735	-0.003	0.936	-0.046	0.268
CAU_L	0.051	0.220	0.030	0.466	0.009	0.820	0.034	0.406	0.021	0.607
PUT_L	0.036	0.379	0.017	0.680	-0.013	0.745	0.020	0.624	-0.017	0.684
PAL_L	0.033	0.427	-0.005	0.905	-0.021	0.602	-0.004	0.927	-0.020	0.631
THA_L	0.017	0.678	-0.043	0.293	**-0.085**	**0.038***	0.005	0.908	-0.060	0.147
ACCsub_L	-0.051	0.219	-0.037	0.366	-0.038	0.354	-0.034	0.414	-0.035	0.396
ACCpre_L	-0.052	0.204	-0.076	0.064	-0.044	0.284	-0.046	0.262	-0.054	0.186
ACCsup_L	-0.021	0.607	-0.048	0.243	-0.009	0.826	-0.030	0.471	-0.053	0.200
Nacc_L	0.034	0.403	0.006	0.888	-0.002	0.971	0.005	0.902	-0.019	0.648
Nacc_R	0.045	0.271	0.015	0.716	-0.028	0.499	0.017	0.686	-0.014	0.727
ACCsup_R	**-0.085**	**0.039***	**-0.082**	**0.045***	-0.071	0.083	**-0.086**	**0.036***	**-0.097**	**0.018***
ACCpre_R	-0.044	0.280	-0.051	0.220	-0.070	0.087	-0.027	0.513	-0.040	0.337
ACCsub_R	-0.026	0.522	-0.033	0.416	-0.067	0.102	-0.029	0.477	-0.032	0.440
THA_R	0.064	0.119	-0.018	0.656	-0.035	0.389	0.026	0.527	-0.019	0.648
PAL_R	0.035	0.391	0.006	0.883	-0.047	0.255	0.001	0.981	-0.013	0.756
PUT_R	0.038	0.350	0.025	0.539	-0.029	0.479	0.032	0.431	0.000	0.997
CAU_R	0.056	0.173	0.042	0.305	-0.010	0.817	0.022	0.590	0.026	0.531

FFG_R	0.026	0.529	0.022	0.601	0.007	0.868	0.076	0.065	0.019	0.638
PHG_R	0.013	0.744	-0.004	0.923	-0.016	0.703	0.005	0.903	-0.027	0.508
HIP_R	0.023	0.581	-0.019	0.644	-0.038	0.353	-0.012	0.769	-0.057	0.165
PFCmed_R	0.020	0.633	0.028	0.502	0.020	0.625	0.063	0.124	0.042	0.313
OLF_R	0.043	0.291	0.022	0.592	0.003	0.934	0.026	0.523	-0.005	0.912

* Significant results, $P < 0.05$, Uncorrected.

Supplementary Table 6. Spearman correlation analysis of amygdala subregion covariance with total CPZ

	L_AAA		L_CAT		R_AAA		R_CAT		R_Ba	
	r	*P*	*r*	*P*	*r*	*P*	*r*	*P*	*r*	*P*
OLF_L	-0.095	0.149	-0.074	0.266	-0.089	0.176	-0.015	0.825	-0.093	0.161
REC_L	-0.026	0.699	-0.086	0.195	-0.079	0.232	-0.027	0.688	-0.054	0.410
HIP_L	-0.123	0.061	-0.082	0.217	-0.102	0.122	-0.064	0.336	**-0.133**	**0.043***
PHG_L	-0.127	0.054	-0.092	0.161	-0.121	0.067	-0.088	0.181	**-0.168**	**0.011***
CAL_L	-0.096	0.145	-0.070	0.290	-0.085	0.196	-0.087	0.185	-0.002	0.978
CUN_L	**-0.143**	**0.030***	-0.126	0.057	-0.112	0.090	**-0.174**	**0.008***	-0.074	0.262
LING_L	-0.110	0.094	-0.076	0.248	-0.103	0.118	-0.069	0.297	-0.077	0.243
CAU_L	-0.083	0.209	-0.007	0.920	-0.066	0.315	0.041	0.533	0.003	0.961
PUT_L	-0.035	0.595	-0.040	0.548	-0.129	0.050	-0.009	0.891	-0.033	0.621
PAL_L	-0.030	0.656	-0.078	0.240	**-0.181**	**0.006***	-0.047	0.482	-0.085	0.196
THA_L	-0.024	0.715	0.064	0.336	-0.055	0.404	0.074	0.263	-0.014	0.834
ACCsub_L	**-0.182**	**0.006***	**-0.142**	**0.031***	-0.105	0.111	-0.084	0.202	-0.076	0.252
ACCpre_L	-0.114	0.084	-0.099	0.133	-0.052	0.432	-0.025	0.708	-0.014	0.838
ACCsup_L	-0.062	0.345	-0.075	0.257	-0.055	0.405	-0.043	0.520	-0.029	0.658
Nacc_L	-0.125	0.059	-0.071	0.283	-0.129	0.051	-0.021	0.746	-0.105	0.113
Nacc_R	-0.116	0.079	-0.071	0.281	-0.124	0.059	-0.030	0.654	-0.083	0.208
ACCsup_R	-0.107	0.106	-0.129	0.050	-0.096	0.144	-0.079	0.230	-0.052	0.433
ACCpre_R	-0.118	0.073	**-0.138**	**0.037***	-0.067	0.313	-0.054	0.410	-0.049	0.461
ACCsub_R	**-0.177**	**0.007***	**-0.168**	**0.011***	**-0.156**	**0.018***	-0.091	0.170	-0.106	0.107
THA_R	-0.026	0.699	0.078	0.236	0.006	0.929	0.096	0.145	0.019	0.773
PAL_R	-0.067	0.314	-0.083	0.210	**-0.185**	**0.005***	-0.054	0.412	-0.082	0.217
PUT_R	-0.041	0.533	-0.038	0.570	-0.123	0.062	0.001	0.986	-0.026	0.697
CAU_R	-0.070	0.290	-0.001	0.987	-0.053	0.420	0.071	0.285	0.032	0.632

FFG_R	-0.085	0.200	-0.027	0.680	**-0.154**	**0.019***	-0.061	0.354	-0.114	0.084
PHG_R	-0.087	0.187	-0.093	0.158	-0.117	0.077	**-0.130**	**0.048***	**-0.161**	**0.014***
HIP_R	-0.089	0.176	-0.099	0.134	-0.114	0.084	-0.099	0.132	**-0.159**	**0.016***
PFCmed_R	-0.103	0.119	-0.104	0.116	-0.057	0.387	0.015	0.822	-0.019	0.768
OLF_R	-0.116	0.079	-0.082	0.212	-0.096	0.148	-0.048	0.470	-0.114	0.084

* Significant results, $P < 0.05$, Uncorrected.
